# Expression Profiling of Genes Related to Endothelial Cells Biology in Patients with Type 2 Diabetes and Patients with Prediabetes

**DOI:** 10.1155/2016/1845638

**Published:** 2016-10-03

**Authors:** Sara Moradipoor, Patimah Ismail, Ali Etemad, Wan Aliaa Wan Sulaiman, Salma Ahmadloo

**Affiliations:** ^1^Department of Biomedical Sciences, Faculty of Medicine and Health Sciences, Universiti Putra Malaysia, Serdang, Selangor, Malaysia; ^2^Department of Medicine, Faculty of Medicine and Health Sciences, Universiti Putra Malaysia, Serdang, Selangor, Malaysia

## Abstract

Endothelial dysfunction appears to be an early sign indicating vascular damage and predicts the progression of atherosclerosis and cardiovascular disorders. Extensive clinical and experimental evidence suggests that endothelial dysfunction occurs in Type 2 Diabetes Mellitus (T2DM) and prediabetes patients. This study was carried out with an aim to appraise the expression levels in the peripheral blood of 84 genes related to endothelial cells biology in patients with diagnosed T2DM or prediabetes, trying to identify new genes whose expression might be changed under these pathological conditions. The study covered a total of 45 participants. The participants were divided into three groups: group 1, patients with T2DM; group 2, patients with prediabetes; group 3, control group. The gene expression analysis was performed using the Endothelial Cell Biology RT^2^ Profiler PCR Array. In the case of T2DM, 59 genes were found to be upregulated, and four genes were observed to be downregulated. In prediabetes patients, increased expression was observed for 49 genes, with two downregulated genes observed. Our results indicate that diabetic and prediabetic conditions change the expression levels of genes related to endothelial cells biology and, consequently, may increase the risk for occurrence of endothelial dysfunction.

## 1. Introduction

The endothelium lines the blood vessels and controls a wide array of vascular functions along with maintaining vascular homeostasis [1]. Some of the major functions of the endothelial cells include regulating the vessel integrity, vascular tone, vascular growth and remodelling, immune responses, cell adhesion, angiogenesis, inflammatory responses, coagulation and platelet activation, haemostasis, and vascular permeability [2]. Sometimes, due to various factors, the endothelium is not able to maintain vascular homeostasis leading to “endothelial dysfunction” [3]. Substantial clinical and experimental evidence suggests that endothelial dysfunction generally occurs in patients diagnosed with Type 2 Diabetes Mellitus (T2DM), in both the resistance and conduit vessels of the peripheral blood circulation along with the coronary circulation [4, 5] and it is one of the major factors that can contribute to the pathogenesis of micro- and macrovascular diseases in these patients [1]. In fact, previous research indicates that as diabetes progresses in patients, there is an increase in the progression of endothelial dysfunction, ultimately leading to atherosclerosis [6]. Despite many proposed mechanisms for this relationship, the definitive pathogenesis remains unclear, possibly because diabetes patients usually display multiple homeostatic imbalances alongside the typically described hyperglycemia. Hyperglycemia and other risk factors such as insulin resistance, oxidative stress, and proinflammatory factors interact with each other to impair endothelial function in patients with T2DM, and the resulting impairments are irreversible in some circumstances. In addition to impaired vasodilator function, diabetes-associated endothelial dysfunction also includes reduction in anticoagulant properties, increase in platelet aggregation, and elevation of adhesion molecules chemokines and cytokines expression [7]. The progression of vasculopathy is greatly dependent upon the degree of hyperglycemia. It can be named as a major causal factor in the development of endothelial dysfunction in patients with Diabetes Mellitus. There have been various mechanisms discovered that can explain how hyperglycemia leads to diabetic endothelial dysfunction, including increased polyol pathway flux, increased advanced glycation end products (AGE) formation, activation of protein kinase C (PKC) isoforms, and increased hexosamine pathway flux [8]. It has also been shown that endothelial dysfunction is also present in patients showing prediabetic symptoms such as impaired fasting glucose and impaired glucose tolerance [9]. It has to be emphasized that most of studies focus on the cellular and molecular mechanisms involved in occurrence of endothelial dysfunction in diabetes patients and PCR array studies regarding this dysfunction are not well established. This study aims to evaluate the gene expression in the peripheral blood of 84 genes related to endothelial cells biology in clinically documented T2DM or prediabetes patients relative to healthy individuals, in order to identify new genes whose expression might be changed under these pathological conditions. This study is trying to open up new targets to management and prevention of endothelial dysfunction and cardiovascular disease in these pathological conditions.

## 2. Materials and Methods

### 2.1. Study Population

The entire study was approved by the Medical Research Ethic Committee (MREC), Ministry of Health, Malaysia (ref number KKM/NIHSEC/P15-758), and informed written consent was obtained from every subject or his/her legally authorized representative. Forty-five participants (22 men, 23 women), of mean age 48.9 ± 5.71 years, have been recruited for this study. The participants were divided into three age-matched groups based on their medical record files in Hospital Serdang: group 1: clinically documented T2DM patients (*n* = 15), group 2: clinically documented prediabetes patients (*n* = 15), and group 3 (control group): healthy individuals with diabetes-free first-degree relatives (*n* = 15). All participants recruited were confirmed free from any late diabetic complications (such as proliferative retinopathy, consolidated nephropathy, kidney failure, heart disease, and autonomic neuropathy), which could influence the results. The main clinical characteristics of all study populations were recorded based on the subject's medical record files in Hospital Serdang and are presented in Table 1.

Peripheral blood (3 mL) was collected from patients and controls by qualified nurse, preserved in Tempus*™* Blood RNA tubes (Applied Biosystem, USA). The tubes were frozen at −20°C before analysis.

### 2.2. RNA Isolation and cDNA Synthesis

High-quality RNA was extracted using Tempus Spin RNA Isolation Kit, according to the manufacturer's instructions (Applied Biosystem, USA). The quantity and purity of the extracted RNA were analysed using Nanodrop ND-1000 spectrophotometry (Thermo Scientific, USA), before being stored in aliquots at −80°C. All RNA samples were also analysed for integrity and genomic DNA contamination using Bioanalyzer 2100 (Agilent Technologies, Palo Alto, CA). Only samples pure enough (A_260_/A_230_ ratio > 1.8, A_260_/A_280_ ratio = 1.8–2.0), with reasonable concentration (>100 ng/*μ*L) and RNA Integrity Number (RIN) ≥8.0, were used as templates for cDNA synthesis. First-strand complementary DNA was synthesized from total RNA (0.8 *μ*g) using the RT^2^ First-Strand Kit (cat # 330401, Qiagen, Germany). The reverse transcription reaction was performed at 37°C. In brief, 0.8 *μ*g of total RNA was added to 2 *μ*L of Buffer GE (5x gDNA Elimination Buffer), and the final volume was made up to 10 *μ*L with RNase-free water. The mixture was denatured at 42°C for 5 min and then immediately cooled by placing on ice for 1 min. Reverse transcription was performed after adding 10 *μ*L of reverse transcription mix to the solution. The reaction mixture was incubated at 42°C for 15 min, after which it was terminated by heating at 95°C for 5 min. The cDNA samples generated (20 *μ*L) were then diluted with 91 *μ*L RNase-free water and stored at −20°C until further analysis.

### 2.3. Gene Expression Profiling

Real-time PCR was carried out by using a Rotor-Gene 6000 Real-Time PCR detection system (Qiagen, Germany). Gene expression was examined using the Human Endothelial Cell Biology RT^2^ Profiler*™* PCR Array (cat # 330231, Qiagen, Germany). Expression of 84 different genes, involved in permeability and vascular tone, angiogenesis, endothelial cell activation, and endothelial cell injury, was targeted for detection by real-time PCR. The RT^2^Profiler*™* PCR Array contains built-in primers for 84 tested and 5 housekeeping genes and positive control elements to determine the efficiency of the reverse transcription reaction, performance of the PCR reaction, and detection of genomic DNA contamination. The PCR mixture for 100 reactions contained 1150 *μ*L of SYBR Green ROX FAST Mastermix (Qiagen, Germany), 102 *μ*L cDNA template, and 1048 *μ*L RNase-free water. The PCR reaction mix was added to the wells of the PCR plate in equal amounts (20 *μ*L), and then the real-time PCR cycling program was run. The thermal cycling program recommended by plates manufacturer for Rotor-Gene 6000 was as follows: 10 min at 95°C followed by 40 cycles: denaturation at 95°C for 15 s, with 30 s annealing and elongation at 60°C, followed by melting curve analysis. The software version 2.1.0 (Qiagen, Germany) was used for analysis.

### 2.4. Data Analysis

To determine the significant differences in the clinical characteristics between the three groups, the Wilcoxon test was carried out, with a significant difference of *p* < 0.05. Data is expressed as the mean ± standard deviation.

The values of Cycle Threshold (C_T_), obtained by real-time PCR experiments were used to calculate the relative changes in gene expression accordingly to 2^−ΔΔCT^ method. B2M and RPLP0 were chosen from the group of five House Keeping Genes (HKG) as the best and least varying reference gene to normalize the gene expression data in order to increase the reliability of comparative C_T_ method-based gene expression quantification. Changes in the gene expression level for evaluated genes were assessed for case groups in relation to the control group with gene expression level set up arbitrarily as 1. The differentially expressed genes, with fold regulation greater than ±3 with *p* < 0.05, are emphasized in this study. Data analyses were performed using the web-based PCR Array data analysis software version 3.5, available at (http://pcrdataanalysis.sabiosciences.com/pcr/arrayanalysis.php).

### 2.5. Pathway Analysis

To determine if any regulatory mechanisms exist between dysregulated genes, we performed in silico analysis using the GNC Pro online analysis tool (http://gncpro.sabiosciences.com/gncpro/gncpro.php).

## 3. Results

Our results demonstrated significantly higher fasting glucose, HbA1c, LDL, triglyceride, and cholesterol in T2DM patients as compared to the control group. The significant difference was also detected in the level of fasting glucose, HbA1c, LDL, and triglyceride between prediabetes and control. We also compared various haematological indices between case groups and control. There was a significantly higher total white blood cell count, neutrophils, and lymphocytes in T2DM patients compared to control (Table 1).

**Figure 1:**
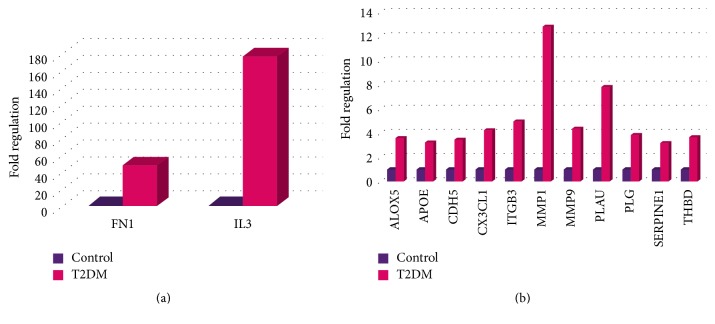
Fold regulation in gene expression in T2DM compared to healthy individuals. Genes with altered expression more than 40-fold in T2DM patients (a). Genes with altered expression more than 3-fold but less than 40-fold in T2DM patients (b).

**Figure 2:**
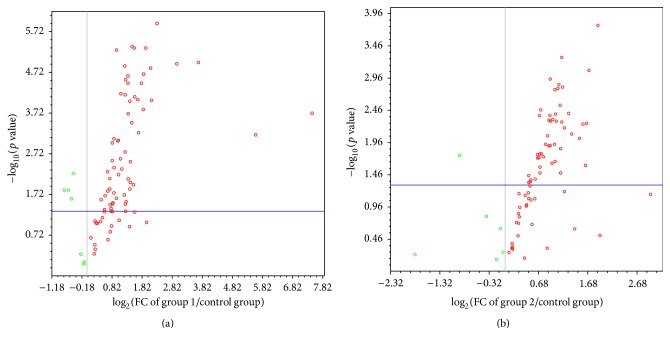
Relative expression comparison for 84 endothelial cells-related gene between cases groups and control group. Volcano plot analysis applied to the PCR Array data revealed 63 genes significantly expressed (*p* < 0.05 with FC ≥ 1 (up or down)) in diabetic patients (a) and 51 genes significantly expressed (*p* < 0.05 with FC ≥ 1 (up or down)) in prediabetic patients (b) compared to healthy individuals. The plot shows a log 2-fold change in gene expression between the two groups on the *x*-axis and the negative log of *t*-test *p* values on the *y*-axis. Each gene is represented by a single point.

In this study, we have used the Human Endothelial Cell Biology RT^2^ Profiler*™* PCR Array, to examine the expression in the peripheral blood of 84 genes, related to biology of endothelial cells, in patients with T2DM and in those with prediabetes relative to healthy individuals. This PCR Array includes representative genes from various biological pathways: angiogenesis, vasoconstriction and vasodilation, inflammatory response, apoptosis, cell adhesion, coagulation, and platelet activation. In case of T2DM, 59 genes showed a significant upregulation. Decreased expression was observed for 4 genes. The significant differences observed in the gene expression profiles in patients with T2DM are presented in Table 2. A majority of these differentially expressed genes were dysregulated less than 3-fold in their expression. Genes ALOX5, APOE, CDH5, CX3CL1, FN1, IL3, ITGB3, MMP1, MMP9, PLAU, PLG, SERPINE1, and THBD were upregulated by greater than 3-fold in their expression (Figure 1). IL3 and FN1 were the most upregulated genes with more than 40-fold changes in their expression (Figure 1(a)). The overall changes observed in the gene expression patterns for T2DM patients are represented in the form of a “volcano plot” wherein Log 2-transformed fold changes in gene expression are plotted against the Student *t*-test *p* values (Figure 2(a)). The genes that are plotted further away from the central axes have greater fold changes and *p* values.

**Figure 3:**
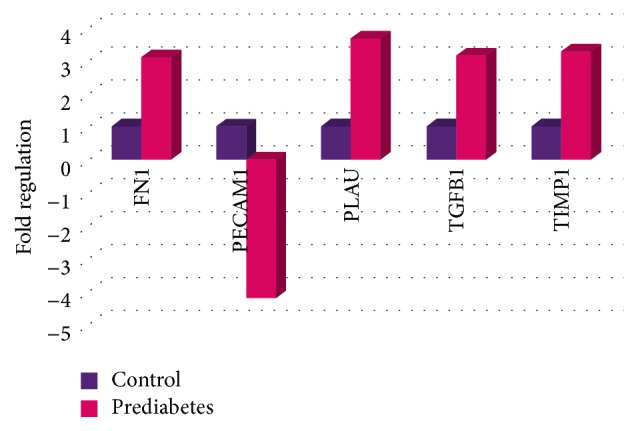
Genes with altered expression more than 3-fold in prediabetic patients compared to healthy individual.

We were able to identify 51 differentially expressed genes in the prediabetic patients (Table 2). PECAM-1 (−4.2076) and SOD1 (−1.8987) were seen to be significantly downregulated and 49 genes were significantly upregulated in these individuals as compared to the control. Genes with dysregulation more than 3-fold in their expression are shown in Figure 3. The overall changes that were observed in the gene expression patterns for prediabetes patients are represented in Figure 2(b) in the form of a volcano plot.

**Figure 4:**
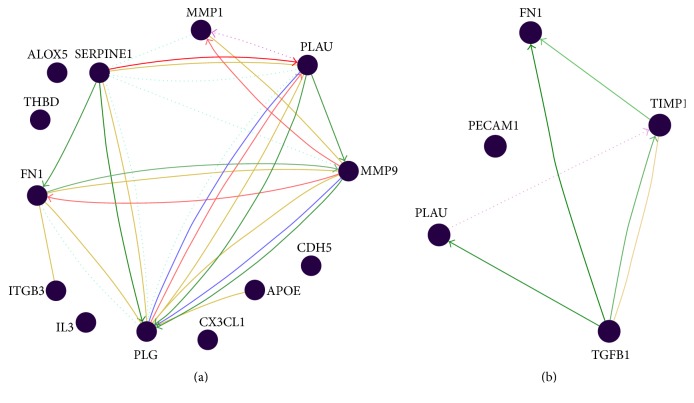
Pathway analysis of genes with altered expression more than 3-fold in T2DM (a) and prediabetes patients (b). The interactions among these genes are represented graphically in this figure. The red line represents downregulation, the green line represents upregulation, the yellow line represents physical interaction, the blue line represents posttranslational modification, the blue dotted line represents predicted protein interaction, and the purple dotted line represents predicted transcription factor regulation.

Pathway mapping in Figure 4 is showing the regulatory mechanisms between genes that were dysregulated more than 3-fold in T2DM and prediabetes patients.

**Figure 5:**
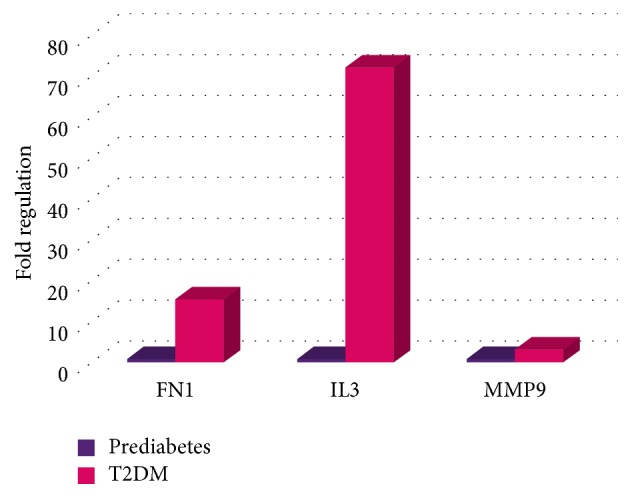
Genes with altered expression more than 3-fold, comparing prediabetic group and T2DM group.

The gene expression levels between diabetic and prediabetic groups were also compared. 17 genes showed significantly greater expression in T2DM relative to prediabetes patients, whereas three genes with a higher expression in prediabetes patients were observed (Table 3). Genes with altered expression more than 3-fold are represented in Figure 5.

## 4. Discussion

The macro- and microvascular pathologies associated with diabetic condition are all characterized by endothelial dysfunction [1]. Most studies focus on the cellular and molecular mechanisms involved in occurrence of endothelial dysfunction in diabetes patients; and gene expression studies regarding this dysfunction in diabetic condition are not well established. This is a first study of its kind, performed in patients with diagnosed T2DM or prediabetes to determine the changes in the blood expression levels of genes related to the biology of endothelial cells. This study is trying to open up new targets in the management and prevention of endothelial dysfunction and cardiovascular disease in these pathological conditions. The expression levels of 84 genes were assessed in both patient groups and the data was compared to their expression levels in the control group. Majority of differentially expressed genes were common for two or more biological processes (Table 4). The alterations in the expression levels of these genes might be associated with the occurrence of endothelial dysfunction in T2DM and prediabetes patients.

Our results appear to provide the first reported data on increased expression levels of IL-3 in patients with T2DM in a whole blood gene expression profiling study. IL-3 is a product of mature T cells and mast cells after activation. Elevated lymphocyte (T cells and B cells) counts were detected in T2DM patients as you can see in Table 1. Moreover, elements of diabetes can directly or indirectly activate T cells [10]. This can be an explanation for overexpression of IL-3 in diabetes patients. The expression of IL-3 was also elevated 2.4413-fold in prediabetes patients which is less than its overexpression in T2DM patients (176.599-fold). Though we do not have any convincing reason for this lower expression of IL-3 in prediabetes patients. IL-3 is classically described as a hematopoietic growth factor. However, it is also known as an endothelial cell activator [11] and can play a role in inflammation [12]. The involvement of IL-3 in inflammatory diseases is supported by its capability to induce expression of adhesion molecules, such as E- and P-selectin, proliferation of endothelial cell, and production of IL-8 [11–13]. Increased IL-3 level in plasma, serum, vitreous, and bone marrow supernatant has been reported in diabetic condition [14–17]. IL-3 is a “good” IL in terms of glucose metabolism, presenting defensive effects in experimental diabetes. Administration of IL-3 twice weekly starting at 2–4 weeks of age delayed the onset and reduced the overall incidence of diabetes in mice. Bone marrow cells obtained from IL-3-treated mice protected other mice from cyclophosphamide-induced diabetes [18]. The uptake of glucose into the cell represents a key point in the regulation of its metabolism and is known to be stimulated by IL-3 [19]. IL-3 regulates glucose uptake by modulating the intrinsic transporting ability of glucose transporters [20]. IL-3 transcriptionally upregulates GLUT1 but also has posttranslational effects on trafficking that are likely to be mediated by Akt and mTOR [21]. On the other hand, IL-3 is believed to play a role in advanced lesions by smooth muscle cell accumulation increasing macrophage activation and neovascularization of the plaque and in the early stages of atherogenesis by facilitating leukocyte extravasation [22]. In spontaneously contracting cultured cardiac myocytes, perfusion with IL-3 induced arrhythmias resulting in a complete cessation of spontaneous contractions and a severe loss of myocyte inotropy; the effects were concentration-dependent and reversible [23]. Thus, overexpression of IL-3 in T2DM patients can have both protective and damaging effects: protective for diabetes and yet damaging for heart and vascular. The major potential for IL-3 in clinical applications is dependent upon its capability to promote the survival, proliferation, and maintenance of hematopoietic progenitor cells [24]. The IL-3 has been used in culture to produce blood cells of various lineages [25]. Moreover, administration of IL-3 to human and primate subjects has enhanced multilineage-hematopoiesis [26, 27]. It increases the number of leukocytes (primarily neutrophils, lymphocytes, and eosinophils), as well as reticulocytes and platelets [28]. However, IL-3 has an inhibitory effect on the expansion of long-term-repopulating hematopoietic progenitor cells and often it was reported that this cytokine can play a negative role in primitive cell expansion [29]. Increased FN1 expression is one of the main features of diabetic angiopathy. Diabetes causes FN1 upregulation in the retina, kidney, heart, and plasma. It can reflect endothelial extracellular matrix changes and, consequently, vessel wall damage in these patients [30, 31]. Diabetes leads to the upregulation of FN1 via an endothelin- (ET-) dependent pathway involving activation of NF-*κ*B and AP-1 transcription factors [31]. According to a study completed by Kanters et al., elevated plasma levels of FN1 may reflect a common pathway of endothelial cell activation in patients with diabetes, which are not found in atherosclerosis without diabetes [30]. Yaghoubi et al. approved the positive association between FN1 level in serum and atherosclerosis progression [32]. According to the results obtained by Rohwedder et al., FN1 can play a dichotomous role in atherosclerosis: while FN worsens the course of atherosclerosis by increasing the atherogenic plaque area, it stabilizes the plaques with fibrous caps and protects from secondary damage and vascular occlusion [33]. The increased MMP-1, MMP-2, and MMP-9 activities and expression induced by high glucose exposure can elevate matrix degradation thereby accelerating atherogenesis and potentially decreasing plaque stability in diabetes [34]. MMP-1 is a biomarker for venous disease and has a role in modulating endothelial permeability by regulation of junctional integrity [35, 36]. T2DM is associated with the elevated blood level of MMP-1 which is positively correlated with coronary heart disease occurrence in these patients [37]. The increase in MMP-9 level and activity may have important consequences for the development of vascular complications associated with diabetes. For instance, the elevated level of MMP-9 in plasma and retinas of diabetic patients can contribute to the development of diabetic retinopathy by altering vascular permeability and capillary cell apoptosis [38, 39]. The proapoptotic role of MMP-9 in hyperglycaemic conditions occurs through the activation of caspase-3 [39]. ALOX5 expression is greater in diabetic compared with nondiabetic plaques and is associated with increased MMP-2 and MMP-9 expression. It has been stated that localized increase in ALOX5 has the potential to cause the acute plaque disruption that precedes the onset of symptoms in both the coronary and cerebral circulations in diabetes [40]. The involvement of ALOX5 in atherosclerosis is not only during the development of atherosclerotic plaques but also during the progression of atherosclerotic plaques toward instability [41]. In this study, we were able to demonstrate that the expression level of the PLAU gene was significantly elevated in the T2DM patients. The same result was reported by Kenichi et al., where they observed increased values for mRNA and protein expressions of PLAU among diabetic rats as compared to control rats [42]. The PLAU is produced by the renal epithelial and the endothelial cells stimulated by the inflammatory cytokines [43]. Hence, chronic inflammation associated with diabetes could be one of the reasons for overexpression of PLAU in diabetes patients. The increased expression of PLAU in the endothelial cells alters the arterial elastic laminae, causes vascular constriction with a narrowing of the lumen, and also enhances the growth of early atherosclerotic lesions [44]. A number of reports have established a direct association between the PLG level or plasmin activity and the occurrence of CAD. To elaborate, two cohort studies with separate perspectives, that is, the FINRISK '92 Haemostasis Study [45] and the Atherosclerosis Risk in Communities Study (ARIC) [46], demonstrate that the PLG level is an independent risk factor for CAD. Integrins have distinct roles in inflammatory cell recruitment to the damaged vessel wall in atherosclerosis [47]. Overexpression of integrins under high glucose concentration can lead to an altered interaction of vascular endothelial cells with their basement membranes, further causing a firmer cell-matrix adhesion [48]. In patients with diabetes integrins induce proangiogenic signalling resulting in aberrant signalling under diabetes that is characteristic of diabetic retinopathy, nephropathy, and macrosomia [7]. According to the results from integrin beta-3 knockout, this integrin is not likely to be a crucial player during development; however, it does mediate pathological neovascularization in adults [49]. ITGB3 plays a prominent role in the angiogenic response in proliferative diabetic retinopathy [50]. According to the results observed in our study, the expression of the ITGA5 gene was found to be significantly upregulated in T2DM patients. A similar result was observed by Stoynev et al., who reported increased levels of ITGA5 in T2DM patients as compared to healthy individuals [51]. An optimal expression of APOE is crucial for maintaining normal metabolism of lipoproteins. Decreased levels of APOE impair the clearance of triglyceride-rich lipoproteins. On the other hand, upregulation of APOE may lead to hypertriglyceridemia through stimulating the production of VLDL triglyceride in the liver and impairing the LPL-mediated lipolysis [52]. It is possible that the nephropathy in T2DM may be associated with the polymorphism of the APOE gene [53]. Vascular endothelial-cadherin is a crucial factor for plaque neovascularization and a subsequent development of plaque instability. Higher expression of CDH5 was observed in complicated plaques and high-grade stenotic lesions [54]. CX3CL1 is a very important factor of atherogenesis, and increased staining of CX3CL1 has been reported in human atherosclerotic coronaries and diabetic vessels [55]. In addition to its role as a chemokine and adhesion molecule, CX3CL1 induces vascular dysfunction by increasing NADPH oxidase-dependent superoxide formation and reduced NO bioavailability [56]. The hyperglycaemia, formation of advanced glycation end products, and cytokine activation in the diabetic condition can induce overproduction of CX3CL1 in the kidneys and aggravate diabetic nephropathy [57]. It has been stated that CX3CL1 can play a prominent role in diabetic renal injury through overproduction of ECM [58]. Zumbach et al. stated that THBD levels were significantly higher in diabetic patients with microvascular complications [59]. These elevations in plasma THBD in diabetic patients are inversely related to protein C activity and positively related to increased markers of thrombin generation, hence constituting a marker of a procoagulant state, reflecting proteolytic injury to the vascular endothelium [60]. In a study conducted by Ewing et al., ANXA5 therapy reduces the vascular inflammation and remodelling, also improving endothelial function in mice [61], thereby indicating its role as a therapeutic potential against atherosclerotic cardiovascular diseases. To the best of our knowledge, no reports in the literature relate the overexpression of ANXA5 to endothelial dysfunction or atherosclerosis. It could be possible that overexpression of this gene protects and limits damage to the target organs in this disease condition. In our study, we have demonstrated that people with T2DM expressed higher levels of TIMP1. Higher levels of TIMP1 expression, in the venous endothelial cell and in plasma, are an early sign of endothelial dysfunction [62]. The same results were observed by Derosa et al., where the authors noticed that the plasma concentration of TIMP1 was significantly increased in T2DM patients, which may cause an abnormal extracellular matrix (ECM) metabolism [63]. The PECAM-1, which is essential for the survival, migration, and functional organization of endothelial cells during vascular development and angiogenesis [64], has been reported to be degraded in the platelets of T2DM patients [65]. The absence of endothelial PECAM-1 resulted in a decreased angiogenesis [66] and might lead to endothelial dysfunction [67].

## 5. Conclusions

Taken together, our findings suggest that the diabetic and prediabetic condition can disrupt the expression of genes involved in the regulation of endothelial cells function and homeostasis. Dysregulation in the expression of these genes can be associated with increased risk for occurrence of endothelial dysfunction in these patients. As endothelial dysfunction appears to be an early indicator of vascular damage, therefore, further research on the expression of genes which can affect endothelial cells function could provide new targets in the management and prevention of macro- and microvascular complications in these pathological conditions. However, all of these data need to be confirmed based on a higher number of patients.

## Figures and Tables

**Table 1:** Clinical characteristics of the participants in the different groups.

	Group 1	Group 2	Group 3
Age (years)	50.33 ± 6.58	47.8 ± 6.24	48.8 ± 4.07
BMI (kg/m^2^)	25.88 ± 1.76	26.97 ± 1.24	26.40 ± 1.21
Fasting glucose (mmol/L)	11.60 ± 2.17^*∗*^	6.52 ± 0.34^*∗*^	4.72 ± 0.33
HbA1c%	12.15 ± 2.01^*∗*^	6.24 ± 0.15^*∗*^	5.00 ± 0.32
HDL (mmol/L)	1.63 ± 0.13	1.72 ± 0.15	1.74 ± 0.56
LDL (mmol/L)	2.22 ± 0.39^*∗*^	2.19 ± 0.38^*∗*^	2.58 ± 0.52
Triglycerides (mmol/L)	1.45 ± 0.23^*∗*^	1.22 ± 0.30^*∗*^	1.02 ± 0.15
Total cholesterol (mmol/L)	4.79 ± 0.83^*∗*^	4.51 ± 0.60	4.26 ± 0.35
WBC (×10^9^/L)	6.17 ± 1.54^*∗*^	5.61 ± 1.33	4.95 ± 1.55
Neutrophils (×10^9^/L)	3.41 ± 1.58^*∗*^	2.54 ± 1.14	2.41 ± 1.11
Lymphocytes (×10^9^/L)	2.14 ± 0.35^*∗*^	1.89 ± 0.91	1.81 ± 0.53
Monocytes (×10^9^/L)	0.46 ± 0.13	0.41 ± 0.17	0.45 ± 0.12
Eosinophils (×10^9^/L)	0.18 ± 0.11	0.19 ± 0.13	0.14 ± 0.12
Basophils (×10^9^/L)	0.04 ± 0.09	0.03 ± 0.06	0.03 ± 0.03

Group 1: T2DM patients; group 2: patients with prediabetes; group 3: control group.

Values shown are the mean ± standard deviation (SD).

*∗* indicates significantly different versus control (*p* < 0.05).

**Table 2:** Genes with altered expression in group 1 and group 2 comparing to group 3.

Symbol	Description	T2DM		Prediabetes	
		Fold changes	95% CI^*∗*^	Fold changes	95% CI^*∗*^
ACE	Angiotensin I converting enzyme	1.69	(0.95, 2.43)	—	—
ADAM17	ADAM metallopeptidase domain 17	1.788	(0.00001, 3.93)	1.5804	(1.01, 2.15)
AGT	Angiotensinogen (serpin peptidase inhibitor, clade A, member 8)	—	—	1.8188	(1.16, 2.47)
ALOX5	Arachidonate 5-lipoxygenase	**3.6158**	(1.93, 5.30)	2.1048	(1.33, 2.88)
ANGPT1	Angiopoietin 1	2.3495	(1.29, 3.41)	—	—
ANXA5	Annexin A5	2.5639	(1.79, 3.34)	1.8502	(1.16, 2.54)
APOE	Apolipoprotein E	**3.2588**	(1.76, 4.76)	—	—
BAX	BCL2-associated X protein	2.5497	(1.69, 3.41)	1.883	(1.21, 2.56)
BCL2	B-cell CLL/lymphoma 2	2.0567	(1.29, 2.82)	1.6294	(1.03, 2.23)
BCL2L1	BCL2-like 1	1.8493	(0.67, 3.03)	—	—
CALCA	Calcitonin-related polypeptide alpha	2.6938	(1.51, 3.88)	1.8562	(1.17, 2.55)
CASP1	Caspase 1, apoptosis-related cysteine peptidase	2.3823	(1.62, 3.14)	2.2207	(1.41, 3.04)
CASP3	Caspase 3, apoptosis-related cysteine peptidase	2.9032	(0.00001, 6.08)	2.8474	(0.00001, 5.98)
CCL2	Chemokine (C-C motif) ligand 2	1.7255	(0.68, 2.77)	—	—
CCL5	Chemokine (C-C motif) ligand 5	0.6962	(0.52, 0.87)	—	—
CDH5	Cadherin 5, type 2 (vascular endothelium)	3.4734	(1.53, 5.42)	—	—
CFLAR	CASP8 and FADD-like apoptosis regulator	2.3999	(1.33, 3.47)	1.5695	(0.74, 2.40)
COL18A1	Collagen, type XVIII, alpha 1	2.4144	(0.85, 3.98)	2.171	(1.13, 3.21)
CX3CL1	Chemokine (C-X3-C motif) ligand 1	**4.2901**	(2.08, 6.50)	2.816	(1.23, 4.41)
EDN1	Endothelin 1	1.8622	(1.11, 2.62)	1.5294	(0.78, 2.28)
EDN2	Endothelin 2	2.456	(0.41, 4.50)	1.3964	(0.96, 1.84)
EDNRA	Endothelin receptor type A	1.9702	(0.81, 3.13)	—	
ENG	Endoglin	—	—	1.9575	(1.25, 2.66)
F2R	Coagulation factor II (thrombin) receptor	—	—	2.1302	(1.16, 3.10)
F3	Coagulation factor III (thromboplastin, tissue factor)	2.7747	(1.53, 4.02)	1.9368	(0.94, 2.93)
FAS	Fas (TNF receptor superfamily, member 6)	2.4166	(1.17, 3.66)	—	—
FASLG	Fas ligand (TNF superfamily, member 6)	—	—	1.6629	(1.03, 2.30)
FGF1	Fibroblast growth factor 1 (acidic)	0.7285	(0.59, 0.87)	—	—
FLT1	Fms-related tyrosine kinase 1	2.177	(1.54, 2.81)	1.4185	(0.99, 1.84)
FN1	Fibronectin 1	**48.4245**	(0.00001, 105.64)	**3.0987**	(1.00, 5.20)
ICAM1	Intercellular adhesion molecule 1	—	—	1.8786	(1.17, 2.58)
IL11	Interleukin 11	2.1411	(1.31, 2.97)	—	—
IL1B	Interleukin 1, beta	2.9739	(1.87, 4.08)	1.8899	(1.24, 2.54)
IL3	Interleukin 3 (colony-stimulating factor, multiple)	**176.599**	(0.00001, 474.01)	2.4413	(1.44, 3.44)
IL6	Interleukin 6 (interferon, beta 2)	1.6806	(0.97, 2.39)	—	—
IL7	Interleukin 7	1.3835	(0.71, 2.06)	—	—
ITGA5	Integrin, alpha 5 (fibronectin receptor, alpha polypeptide)	2.6864	(1.61, 3.76)	1.8891	(1.19, 2.59)
ITGAV	Integrin, alpha V (vitronectin receptor, alpha polypeptide, antigen CD51)	1.7913	(1.17, 2.41)	1.6077	(1.00, 2.21)
ITGB1	Integrin, beta 1 (fibronectin receptor, beta polypeptide, antigen CD29 including MDF2, MSK12)	1.9693	(1.44, 2.49)	1.6144	(0.98, 2.24)
ITGB3	Integrin, beta 3 (platelet glycoprotein IIIa, antigen CD61)	**4.9899**	(2.90, 7.08)	2.5427	(0.65, 4.43)
KDR	Kinase insert domain receptor (a type III receptor tyrosine kinase)	1.6884	(0.78, 2.59)	—	—
KIT	V-kit Hardy-Zuckerman 4 feline sarcoma viral oncogene homolog	1.6025	(0.89, 2.31)	1.6211	(1.07, 2.18)
MMP1	Matrix metallopeptidase 1	**12.8082**	(3.18, 22.43)		
MMP2	Matrix metallopeptidase 2	2.6715	(0.87, 4.47)	1.7691	(1.13, 2.41)
MMP9	Matrix metallopeptidase 9	**4.4005**	(2.21, 6.60)	—	—
NOS3	Nitric oxide synthase 3 (endothelial cell)	2.5532	(1.62, 3.48)	—	—
PECAM1	Platelet/endothelial cell adhesion molecule	0.5887	(0.35, 0.82)	**0.2377**	(0.14, 0.33)
PF4	Platelet factor 4	—	—	1.397	(0.62, 2.17)
PGF	Placental growth factor	2.9547	(1.87, 4.04)	2.2258	(1.21, 3.24)
PLAT	Plasminogen activator, tissue	1.788	(1.03, 2.55)	2.0088	(1.03, 2.99)
PLAU	Plasminogen activator, urokinase	**7.8263**	(2.73, 12.93)	**3.6782**	(1.53, 5.83)
PLG	Plasminogen	**3.8718**	(1.36, 6.38)	2.18	(0.52, 3.84)
PROCR	Protein C receptor, endothelial	—	—	1.7096	(1.08, 2.34)
PTGIS	Prostaglandin I2 (prostacyclin) synthase	—	—	2.18	(1.22, 3.14)
PTGS2	Prostaglandin-endoperoxide synthase 2 (prostaglandin G/H synthase and cyclooxygenase)	2.5639	(1.08, 4.05)	2.0312	(1.25, 2.81)
PTK2	PTK2 protein tyrosine kinase 2	1.7263	(0.84, 2.62)	—	—
SELE	Selectin E	1.8021	(0.90, 2.70)	2.3075	(1.17, 3.44)
SELL	Selectin L	2.4044	(1.58, 3.22)	1.6476	(1.16, 2.13)
SELPLG	Selectin P ligand	2.42	(1.80, 3.04)	2.0023	(1.37, 2.64)
SERPINE1	Serpin peptidase inhibitor, clade E (nexin, plasminogen activator inhibitor type 1), member 1	**3.2035**	(1.87, 4.53)	2.0463	(1.12, 2.97)
SOD1	Superoxide dismutase 1, soluble	0.6517	(0.46, 0.84)	0.5267	(0.33, 0.73)
SPHK1	Sphingosine kinase 1	2.2258	(1.35, 3.10)	1.6339	(0.65, 2.62)
TEK	TEK tyrosine kinase, endothelial	—	—	2.2454	(1.29, 3.20)
TFPI	Tissue factor pathway inhibitor (lipoprotein-associated coagulation inhibitor)	1.4959	(0.95, 2.04)	—	—
TGFB1	Transforming growth factor, beta 1	2.8056	(1.88, 3.73)	**3.142**	(1.70, 4.58)
THBD	Thrombomodulin	**3.685**	(2.00, 5.37)	2.0097	(1.20, 2.82)
THBS1	Thrombospondin 1	1.8588	(1.18, 2.54)	1.433	(0.75, 2.12)
TIMP1	TIMP metallopeptidase inhibitor 1	2.6814	(1.82, 3.54)	**3.283**	(1.82, 4.74)
TNF	Tumor necrosis factor	1.5922	(1.11, 2.07)	—	—
TNFSF10	Tumor necrosis factor (ligand) superfamily, member 10	1.5084	(1.07, 1.94)	—	—
TYMP	Thymidine phosphorylase	—	—	2.9739	(1.01, 4.93)
VEGFA	Vascular endothelial growth factor A	2.0406	(1.44, 2.64)	—	—
VWF	Von Willebrand factor	2.035	(1.41, 2.66)	1.6226	(0.92, 2.32)

Group 1: T2DM patients; group 2: patients with prediabetes; group 3: control group.

^*∗*^95% confidence interval.

**Table 3:** Direct comparison of gene expression levels between groups 1 and 2.

Gene	Description	Fold changes	95% CI^*∗*^
ADAM17	ADAM metallopeptidase domain 17	1.1313	(0.00001, 2.49)
ALOX5	Arachidonate 5-lipoxygenase	1.7179	(0.85, 2.59)
APOE	Apolipoprotein E	2.2932	(1.15, 3.44)
EDN2	Endothelin 2	1.7589	(0.24, 3.28)
EDNRA	Endothelin receptor type A	1.8643	(0.97, 2.76)
FGF1	Fibroblast growth factor 1 (acidic)	0.6051	(0.44, 0.77)
FLT1	Fms-related tyrosine kinase 1	1.5347	(1.04, 2.03)
FN1	Fibronectin 1	**15.6273**	(0.00001, 34.83)
IL11	Interleukin 11	2.4116	(1.37, 3.45)
IL1B	Interleukin 1, beta	1.5735	(0.93, 2.22)
IL3	Interleukin 3	**72.3372**	(0.00001, 194.69)
MMP2	Matrix metallopeptidase 2	1.5101	(0.52, 2.50)
MMP9	Matrix metallopeptidase 9	**3.2641**	(1.58, 4.95)
NOS3	Nitric oxide synthase 3 (endothelial cell)	2.1347	(1.37, 2.90)
PECAM1	Platelet/endothelial cell adhesion molecule	2.4771	(1.67, 3.28)
PLAU	Plasminogen activator, urokinase	2.1278	(1.14, 3.11)
PTGIS	Prostaglandin I2 (prostacyclin) synthase	0.4289	(0.23, 0.63)
SELL	Selectin L	1.4594	(0.90, 2.02)
THBD	Thrombomodulin	1.8336	(1.19, 2.48)
TYMP	Thymidine phosphorylase	0.4013	(0.13, 0.67)

Group 1: patients with type 2 diabetes; Group 2: patients with prediabetes.

^*∗*^95% confidence interval.

**Table 4:** Functional gene grouping.

Angiogenesis	ANGPT1, CCL2, CCL5, CX3CL1, EDN1, EDNRA, ENG, F3, FASLG, FGF1, FLT1, FN1, IL1B, IL6, ITGA5, ITGAV, ITGB1, ITGB3, KDR, KIT, MMP2, MMP9, NOS3, PF4, PGF, PLAU, PTGS2, SERPINE1, SPHK1, TEK, THBS1, TYMP, VEGFA.

Vasoconstriction & vasodilation	ACE, AGT, ALOX5, APOE, CALCA, CX3CL1, EDN1, EDN2, EDNRA, F2R, ICAM1, NOS3, PTGIS, PTGS2, SOD1.

Inflammatory response	ACE, AGT, ALOX5, APOE, CALCA, CCL2, CCL5, CX3CL1, EDNRA, F2R, F3, FN1, IL1B, IL6, PTGS2, SELE, SPHK1, TGFB1, THBS1, TNF.

Apoptosis	ANXA5, BAX, BCL2, BCL2L1, CASP1, CASP3, CCL2, CCL5, CFLAR, CX3CL1, EDN1, EDNRA, FAS, FASLG, IL1B, IL3, IL6, IL7, PF4, PTK2, SPHK1, TEK, THBS1, TNF, TNFSF10.

Cell adhesion	ADAM17, AGT, BCL2, CALCA, CDH5, COL18A1, CX3CL1, ENG, FGF1, FN1, ICAM1, IL1B, ITGA5, ITGAV, ITGB1, ITGB3, KDR, PECAM1, PLAU, PLG, PTK2, SELE, SELL, SELPLG, SERPINE1, TGFB1, THBS1, TNF, VEGFA, VWF.

Coagulation	ANXA5, EDN1, F2R, F3, FN1, MMP1, PECAM1, PF4, PLAT, PLAU, PLG, PROCR, PTK2, SELL, SELPLG, SERPINE1, TEK, TFPI, THBD, THBS1, TIMP1, VWF.

Platelet activation	APOE, CX3CL1, F2R, FN1, IL11, IL6, ITGB3, NOS3, PECAM1, PF4, PLG, SERPINE1, SOD1, TGFB1, THBD, THBS1, TIMP1, VEGFA, VWF.
