# Erratum to “Expression Profiling of Genes Related to Endothelial Cells Biology in Patients with Type 2 Diabetes and Patients with Prediabetes”

**DOI:** 10.1155/2017/9764930

**Published:** 2017-02-15

**Authors:** Sara Moradipoor, Patimah Ismail, Ali Etemad, Wan Aliaa Wan Sulaiman, Salma Ahmadloo, Somayeh Khazaei

**Affiliations:** ^1^Department of Biomedical Sciences, Faculty of Medicine and Health Sciences, Universiti Putra Malaysia, Serdang, Selangor, Malaysia; ^2^Department of Medicine, Faculty of Medicine and Health Sciences, Universiti Putra Malaysia, Serdang, Selangor, Malaysia

In the article titled “Expression Profiling of Genes Related to Endothelial Cells Biology in Patients with Type 2 Diabetes and Patients with Prediabetes” [1], Dr. Somayeh Khazaei was missing from the authors' list. The corrected authors' list is shown above.
